# Opportunities and challenges: interleukin-22 comprehensively regulates polycystic ovary syndrome from metabolic and immune aspects

**DOI:** 10.1186/s13048-023-01236-9

**Published:** 2023-07-31

**Authors:** Yuli Geng, Zhuo Liu, Runan Hu, Wenwen Ma, Xiao Wu, Haoxu Dong, Kunkun Song, Xiaohu Xu, Yanjing Huang, Fan Li, Yufan Song, Mingmin Zhang

**Affiliations:** 1 Institute of Integrated Traditional Chinese and Western Medicine, Tongji Hospital, Tongji Medical College, Huazhong University of Science and Technology, 1095 Jiefang Avenue, Wuhan, Hubei 430030 China; 2 Department of Traditional Chinese Medicine, Tongji Hospital, Tongji Medical College, Huazhong University of Science and Technology, 1095 Jiefang Avenue, Wuhan, Hubei 430030 China

**Keywords:** PCOS, IL-22, Metabolism, Treatment, Mechanism

## Abstract

Polycystic ovary syndrome (PCOS) is known as a prevalent but complicated gynecologic disease throughout the reproductive period. Typically, it is characterized by phenotypic manifestations of hyperandrogenism, polycystic ovary morphology, and persistent anovulation. For now, the therapeutic modality of PCOS is still a formidable challenge. Metabolic aberrations and immune challenge of chronic low-grade inflammatory state are significant in PCOS individuals. Recently, interleukin-22 (IL-22) has been shown to be therapeutically effective in immunological dysfunction and metabolic diseases, which suggests a role in the treatment of PCOS. In this review, we outline the potential mechanisms and limitations of IL-22 therapy in PCOS-related metabolic disorders including its regulation of insulin resistance, gut barrier, systemic inflammation, and hepatic steatosis to generate insights into developing novel strategies in clinical practice.

## Introduction

Polycystic ovary syndrome (PCOS) is a systematic endocrine disorder that negatively impacts the overall health of reproductive-aged females [1]. The incidence of PCOS varies from 6 to 10% worldwide according to different diagnostic criteria [2]. The characteristics of PCOS are featured as hyperandrogenism, ovulatory abnormalities, and morphologically polycystic ovary [3]. Although other pathological manifestations are excluded from the diagnosis criteria, the systemic metabolic dysfunction and chronic low-grade inflammatory state are predominant in PCOS patients, including insulin resistance (IR), dyslipidemia, central obesity and so on [4, 5]. There is accumulating evidence that PCOS women have an increased risk of developing metabolic syndrome (MS), type 2 diabetes mellitus (T2DM), and cardiovascular diseases [6]. PCOS is considered the leading cause of infertility due to anovulation [7]. And it poses a great threat to women’s long-term physical and mental health [8–10].

Due to the complicated etiology and pathogenesis of the endocrine-metabolic disorders behind PCOS, a more effective treatment has yet to be developed [11, 12]. Despite that lifestyle intervention has been suggested as the primary line of therapy for PCOS, it appears inadequate and unsatisfactory when it comes to the involvement, compliance, and constancy [3]. Generally, the assisted pharmacological treatment of PCOS includes combined oral contraceptive pills (COCPs), insulin-sensitizing agents, anti-androgen and anti-obesity pharmacological agents [3]. Oral contraceptive (OC) therapy can ameliorate hyperandrogenism and restore menstrual patterns, however, it is not appropriate for patients with reproductive requirements [12, 13]. Long-term use of OC therapy will also bring side effects of circulatory disorders comprising venous thrombosis and hypertension [13–18]. Insulin-sensitizing agents, represented by metformin, are beneficial for alleviating insulin resistance, menstrual irregularities, hirsutism, anovulation, and obesity [19]. Although it shows positive impacts in multiple aspects, the efficacy of metformin is still limited compared with the first-line management and the gastrointestinal adverse effects are common [20, 21]. Oral ovulation induction agents including letrozole, clomiphene citrate, and metformin are prescribed to treat subfertile women with PCOS that are seeking pregnancy [3]. Other therapies are all effective to varying degrees but still have restrictions. Researchers have been dedicated to searching for more effective substitutions. Recently, a cytokine called interleukin-22 (IL-22) has aroused attention by virtue of its advantages in multiple models of metabolic diseases [22–24]. The effects of IL-22 in modulating metabolism were initially identified in hepatic steatosis [25]. Subsequently, it has also been found to ameliorate symptoms in a number of classical metabolic diseases like T2DM, MS, and obesity [22, 23, 26]. Nowadays, the research of IL-22 has been further expanded to treatments in PCOS models, which demonstrates regulatory functions in restoring hormones, ovarian morphologies, estrous cycles, and pup numbers in PCOS models [27–29]. Although certain extent of consensus has been established, there are conflicting and inconsistent findings regarding IL-22 therapy in metabolic disorders. Therefore, this review will thus concentrate on the benefits and contradictions of IL-22 in relation to metabolic and immune impairment in PCOS. We anticipate providing evidence for the future application in clinical practice and identifying difficulties that demand prompt solution.

## Overview of IL-22

IL-22 is a member of the IL-10 family of cytokines, along with IL-19, IL-20, IL-24, IL-26, type III interferon (IFN) group, and others. [30, 31] It is a cytokine with an alpha helix that is specifically generated by lymphoid lineage cells, such as T cells, natural killer T (NKT) cells, and innate lymphocyte cells (ILCs) [32]. T-helper 17 (Th17) cells are the predominant generator of IL-22 in rodents, whereas T-helper 22 (Th22) cells are the primary source in humans [33–35]. The IL-22 receptor (IL-22R) is composed of two distinct subunits, IL-10R2 and IL-22R1, which are responsible for transmitting signals from IL-22 [32, 36]. On account of the ubiquitous expression of IL-10R2, the cellular sensitivity to IL-22 is mostly dependent on the IL-22R1 expression [37]. IL-22R1 is highly detected in multiple tissues and organs including epidermis, liver, kidney, gastrointestinal and respiratory systems but except immune cells [37]. IL-22-binding protein (IL-22BP) is another receptor that competitively inhibits IL-22 from interacting with IL-22R complex thus neutralizing its activity [38]. In inflammatory diseases, IL-22 may either contribute to the development or act as a buffer against their progression [39]. On the one hand, it has the potential to transduce inflammatory signals in inflamed tissues and stimulate the synthesis of pro-inflammatory effectors [40]. On the other hand, it takes part in antimicrobial defense, injury repairment and tissue regeneration [41, 42]. With the advancement of the research, the function of IL-22 has expanded beyond inflammatory and auto-immune diseases and spawned a new surge in endocrine-metabolic disorders [22, 26].

## Core pathogenesis of PCOS

**Fig. 1:**
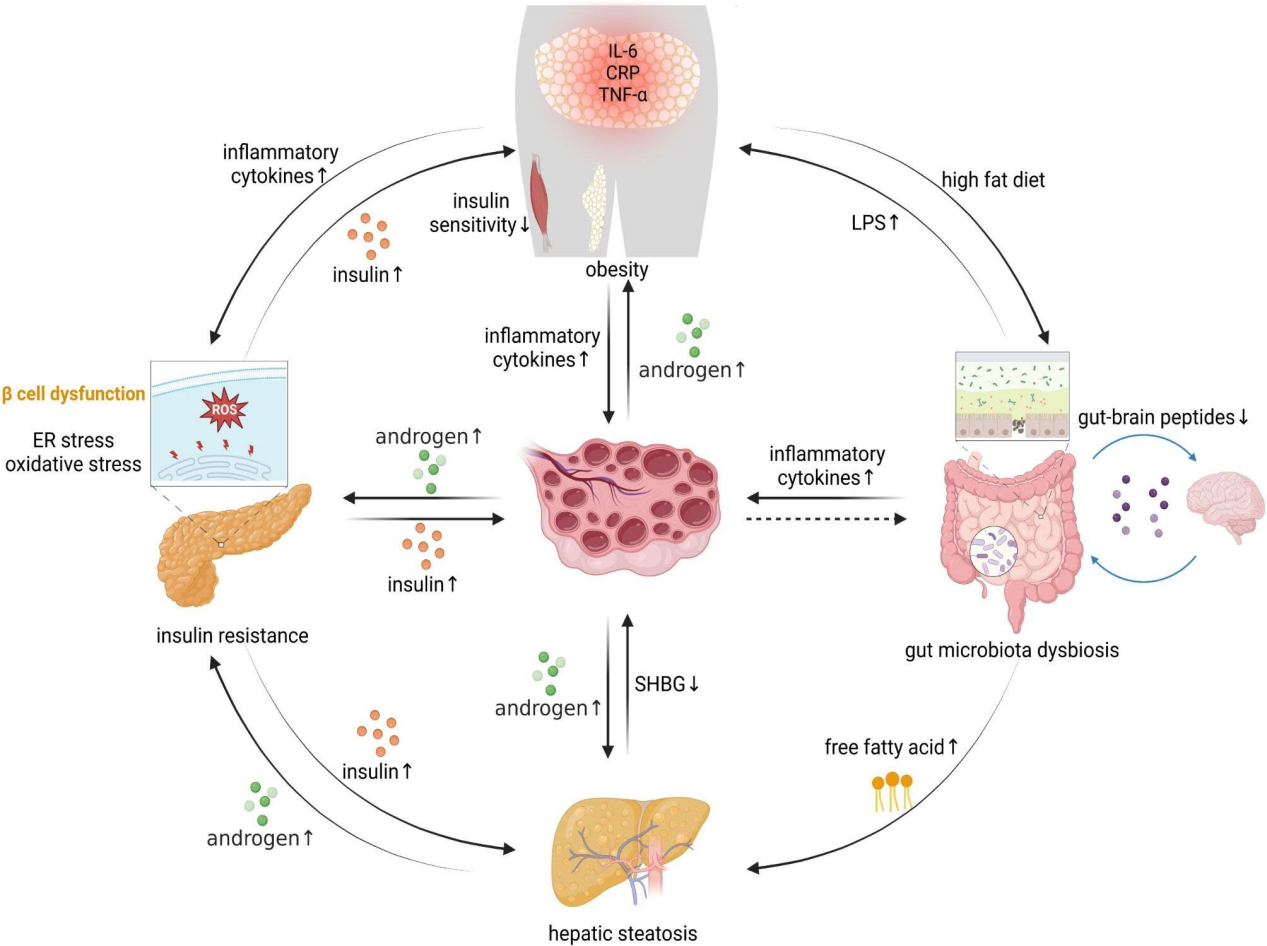
PCOS related metabolic disorders and immunity impairments

Although environment and genetics are implicated in the incidence of PCOS, hyperandrogenemia, IR, and adipose tissue dysfunction are central to the progression of PCOS [43] (Fig. 1). Androgen excess is considered as a critical feature in a majority of PCOS women [44], which will contribute to masculinizing features and follicular arrest [45]. IR is another significant manifestation which exists in approximately 50% PCOS patients regardless of obesity [46, 47]. IR exacerbates hyperandrogenism by stimulating androgen synthesis and inhibiting sex hormone-binding globulin (SHBG) [48, 49]. The interaction between IR and hyperandrogenism compromises ovum growth, endometrial receptivity, the neuroendocrine of adrenal glands and ovaries [50]. Apart from the reproductive failure, IR also affects lipid metabolism in PCOS [51]. Visceral obesity increases the likelihood of metabolic aberrations and reproductive abnormalities [52]. It can also result in oxidative stress and chronic inflammation in general and in specific tissues [53, 54]. According to the available data, the level of inflammatory mediators rises in PCOS patients which also leads to ovarian dysfunction [55]. Recently, gut microbiota alteration has also been confirmed as closely relevant to PCOS accompanying the increasing epithelial permeability and leakage of inflammatory cytokines to circulation which will promote the inflammatory status [56–61]. Nonalcoholic fatty liver disease (NAFLD), another metabolic complication, has been indicated an interplay with PCOS in depth. NAFLD encompasses a spectrum of pathological manifestations, ranging from common hepatic fat deposition to nonalcoholic steatohepatitis (NASH), which might further progress to cirrhosis [62]. It shares many pathophysiologic mechanisms with PCOS, of which the most significant is IR and hyperandrogenism [63].

Hyperandrogenemia is a significant hallmark in PCOS individuals which will exacerbate the reproductive plight. IR could interact with excess androgen and impact lipid metabolism, thus further impairing the fertile capacity. The inflamed central adiposity contributes to systemic inflammation and ovarian dysfunction. Alterations in gastrointestinal microbiota and NAFLD have also been associated with PCOS, promoting inflammation and IR correspondingly.

## IL-22 and IR

Insulin, an indispensable hormone generated by pancreatic beta cells, signals through transmembrane receptor in response to the elevation of glucose in plasma [64, 65]. Women with PCOS have been observed impaired insulin sensitivity in peripheral tissue compared to normal females [66]. Hyperinsulinemia is a follow-up adaptive regulation to ensure the maintenance of normoglycemia [67]. The underlying mechanism of insulin resistance is still uncertain, but skeletal muscle and adipose tissue have been shown to display insulin signaling defects [68, 69]. Reduced expression of glucose transporter type 4 (GLUT-4) in lipocytes might also be a reason of impaired insulin responsiveness [66, 70]. β-cell dysfunction is another culprit of IR which militates against proinsulin maturation and insulin secretion [71]. However, as IR has a genetic predisposition in PCOS, it remains dubious whether the defects in β-cell function precede IR or develop after it [72–74]. Furthermore, the interior milieu of PCOS contributes to the aggravation of IR, especially the excess androgens. Androgen, for one thing, can modify the secretion of adipokines and promote visceral fat accumulation hence inhibiting insulin sensitivity in adipose tissue and skeletal muscles [75]. For another thing, excessive insulin inhibits SHBG formation in the liver and promotes androgen release, thus resulting in the elevation of free testosterone (T) [76, 77]. The reciprocity between IR and hyperandrogenemia forms a vicious circle, leading to the aggravation of PCOS [78–80].

### The innerpancreatic effects of IL-22

**Fig. 2:**
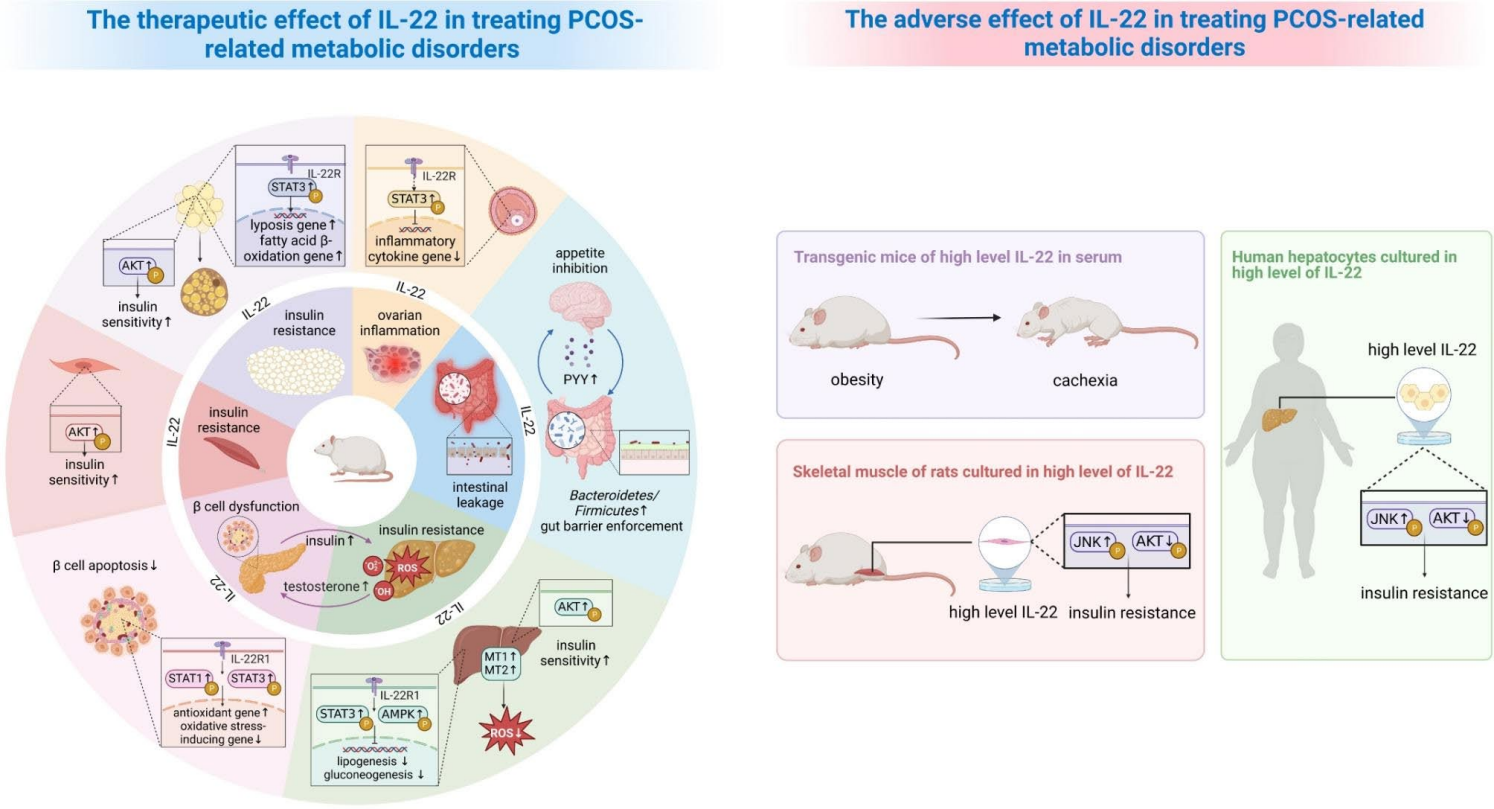
IL-22 may alleviate PCOS-related metabolic disorders and immunity impairments

According to recent research, the entire pancreas contains IL-22-producing cells [81]. Hasnain et al. declared that islets from mice given a high fat diet (HFD) displayed more severe endoplasmic reticulum (ER) stress and oxidative stress than islets from mice of normal chow [23] (Fig. 2). The findings in human beta cells were identical to those observed in vivo when IL-22 was supplemented [23]. Specifically, IL-22 inhibited the apoptosis of β cells, thus restoring insulin secretion and improving insulin sensitivity in obese mice [22, 23]. IL-22 took effect by upregulating antioxidant genes and inhibiting oxidative stress-inducing genes mediated by signal transducer and activator of transcription 1 (STAT1) and STAT3 [23]. They also indicated that IL-22 might alternatively signal through IL-22R1 ligand as the blockade of IL-22R1 signaling elicited ER stress in β cells [23]. Wang et al. confirmed that IL-22R1-deficient mice developed severe adiposity and insulin resistance while no differences were observed in IL-22-deficient mice. Another new study also found enhanced serous IL-22 triggered IL-22R1/Janus kinase 1 (JAK1) /STAT3 signaling pathway in islets, which improved insulin resistance in PCOS rats [29].In addition, Park et al. set a cohort of transgenic mice IL-22TG6 with serous IL-22 at a moderate level (~ 600 pg/ml) to mimic the treatment of IL-22 [82]. However, they argued that wildtype and IL-22TG6 mice showed no appreciable variations in either glucose tolerance or insulin sensitivity with normal diet or HFD. In parallel, they observed no improvement in insulin resistance in HFD mice following a long-term and low-dose administration of recombinant mouse IL-22 (rmIL-22). This result is corresponding with the findings of Yang et al. but contrary to the findings of Hasnain’s study, despite using the same agentia [23, 25, 82]. Surprisingly, Park et al. also contradicted the notion that the strong STAT3 phosphorylation occurred in acinar cells rather than β cells in acute pancreas injury models [82]. These opinions are provocative and the role of IL-22, including its target in the pancreas, an appropriate administration dosage, and other mechanisms of its efficacy, needs to be elucidated in detail.

### The extrapancreatic effects of IL-22

#### IL-22 promotes adipose tissue browning

Over the last decades, emphasis has been placed on the role of brown adipose tissue (BAT) in increasing the metabolic rate and alleviating IR [83–85]. The transplantation of BAT to rodents with PCOS has been shown to improve critical characteristics [86]. Qi et al. pointed out that IL-22 was capable of promoting white fat browning in PCOS mice, accompanied by a dramatical elevation of the thermogenic markers in subcutaneous adipose and brown adipose [27, 28]. They proposed that the promotion of adipose tissue browning represented a potential mechanism by which IL-22 facilitates IR in PCOS [27, 28]. Hasnain et al. also found a greater distribution of brown fat in obese rodents treated with IL-22 [23].

By promoting white fat browning and reducing oxidative and ER stress in β cells, IL-22 may be able to enhance insulin sensitivity and secretion. In addition, IL-22 therapy modulates chronic low-grade inflammation through ameliorating obesity and attenuating local inflammatory state in granulosa cells. Furthermore, exogenous IL-22 may alter the gut flora community via reinforcement of gut mucosal barrier and decrease of LPS leakage. Gut-brain axis may also be involved in the regulation of body weight. IL-22 significantly activates the STAT3 pathway, which prevents the hepatic lipogenesis and gluconeogenesis. Furthermore, IL-22 upregulates MT1 and MT2 to reduce oxidative stress in hepatocytes. High blood levels of IL-22, however, appear to have negative consequences such as cachexia and IR.

#### Does IL-22 induce IR in peripheral organization?

After administration with IL-22 fragment crystallizable (IL-22Fc), Wang et al. found that increased protein kinase B (AKT) phosphorylation of insulin-targeted peripheral organization in diet-induced obese (DIO) mice was in support of improved insulin responsiveness [22]. In contrast, a study demonstrated that IL-22 inhibited insulin from stimulating glucose absorption in vitro incubation of rats’ muscles [87]. Similar effects were observed in human hepatocytes, displaying a decreased level of AKT phosphorylation and an attenuated glucose metabolism in response of insulin challenge [87]. Fabbrini et al. showed that pretreatment with IL-22 enhanced c-Jun kinase (JNK) phosphorylation which presumably explained the suppressive effect of insulin by IL-22 [87, 88]. Furthermore, they noticed that obese individuals with insulin resistance exhibited greater polarization and infiltration of CD4^+^ T cells that generated IL-22 in the subcutaneous adipose tissue [87]. Nevertheless, the culture condition in vitro deserves our attention, which respectively set a 7.5 ng/mL of IL-22 for hepatocytes and 100 ng/mL of IL-22 for skeletal muscles [87]. As Hasnain et al. argued, it was unlikely to reach such high concentration in muscles in vivo [23], the hepatocytes probably likewise. This dosage is inapplicable as Park et al. demonstrated that exorbitant IL-22 in plasma may drive the situation to unfavorable consequences [82].

## IL-22 and inflammation

Accumulating evidence uncovers that the sustaining status of chronic low-grade inflammation is a critical manipulator for the disturbance of PCOS where obesity serves as a primary trigger [89]. With a high prevalence up to 38-88% in PCOS, obesity, especially abdominal type, will promote the burden of inflammation, boosting the level of oxidative stress and various inflammatory markers [5]. By enhancing the inflammatory cytokines and recruiting immune cells, the inflamed adipose tissue sustains the state of inflammation [90]. Representative inflammatory mediators are higher in circulation, including tumor necrosis factor-α (TNF-α), IL-6, and C-reactive protein (CRP) [91–94]. These inflammatory cytokines lead to the aggregation of IR and stimulation of androgen production [54]. In addition to the systemic inflammatory state, ovarian tissue is also confronted with inflammation accompanied by increased macrophages and lymphocytes infiltration [92].

Park et al. illustrated that the level of IL-22 is considerably low in serum despite consuming HFD in absence of exogenous inflammatory stimulation [82]. However, Wang and colleagues found a remarkable increase of IL-22 in HFD-fed mice [22]. Moreover, in T2DM patients, the frequency of Th-22 cells is higher than control individuals which is also positively correlated with blood concentration of IL-22 [95, 96]. In metabolically abnormal subjects of obesity, Fabbrini also provided evidence that increased IL-22 in serum and CD4^+^ T cells which produced IL-22 in adipose tissue might be related with the stimulation of cytokines such as IL-1β [87, 97]. Sabat has indicated that the difference between rodents and humans in IL-22 level is due to the fact that humans are exposed to a variety of stressors over the long lifespan [98]. Consequently, the state of inflammation is distinct from that of the rodents in the experimental environment. This clarification appears plausible but may not apply to all the metabolic disorders. Two independent groups observed deficient secretion of IL-22 was both observed in PCOS patients and PCOS-like murine models [28, 99]. This result might pave a way for the IL-22 therapy in experimental studies and clinical trials of PCOS, while a new clinical study observed no differences in the circulating concentrations of IL-22 and IL-22BP between PCOS individuals and healthy controls [100]. Therefore, we need more clinical studies and observations to confirm the alterations in IL-22 levels in patients with PCOS, as well as more effort to investigate the underlying mechanisms of the differences in IL-22 levels among various metabolic diseases.

Furthermore, Wang et al. found IL-22 regulated lipid metabolism by directly activating STAT3 in adipose tissue [22]. After high doses of IL-22-Fc administration (50–100 µg/mouse, twice weekly), the obesity was remarkably improved in DIO and db/db mice [22]. Besides, IL-22Fc increased the expression of genes involved in triglyceride lipolysis and fatty acid β-oxidation, while decreasing the pro-inflammatory gene expressions involved in obesity [22, 101, 102]. However, Park et al. found that considerable high concentration of IL-22 (4000–7000 pg/ml) might cause cachexia, manifesting an abnormally thin phenotype [82]. Yang et al. revealed that long-term therapy with rmIL-22 (300 ng/g, daily for 36 days) did not impact body weight, corroborating the Park’s findings that a relatively high level did not affect obesity [25, 82]. However, another study still concluded that the low-dose treatment (20 ng/g or 100 ng/g, twice weekly for 4 weeks) could ameliorate obesity [23]. Taken together, ambiguity and uncertainty enrich the efficacy and administration scheme of IL-22 in treating obesity, making it imperative to conduct additional studies concentrating on the mechanism and safety.

Ovarian local inflammation is also an important component of the systemic inflammatory situation in PCOS. IL-22 administration markedly decreased inflammatory cytokines and their gene expressions in granulosa cells from PCOS patients [28]. Qi et al. implied that IL-22 might activate STAT3, resulting in an increase of adenosine monophosphate kinase and a reduction in the inflammatory state of macrophages [28]. Therefore, modulation of local inflammation by IL-22 also provides mechanistic insight into regulating ovarian dysfunction.

## IL-22 and gut

Recent studies have put an emphasis on the interaction between gut microbiota dysbiosis and PCOS [57, 58, 103–105]. Several leading studies have highlighted a reduction of α diversity and an alteration of β diversity, the former of which is a causal factor for obesity [57, 58, 106, 107]. The variation of intestinal microbial composition was initially confirmed by Kelly et al. in letrozole-induced PCOS rats, typifying decreased *Bacteroides* and increased *Firmicutes* [56]. The elevation of *Firmicutes* has been found closely connected with the occurrence of classical metabolic diseases such as obesity, T2DM, and MS [108]. In addition, the proportion of probiotics declined both in PCOS models and individuals, such as those in charge of maintaining intestinal integrity by producing short-chain free fatty acids (SCFAs) [109]. These changes tend to trigger the destruction of gut epithelial barrier and the increase in intestinal permeability which contributes to systemic endotoxemia and host immunity activation [78]. Thus, the increased circulating lipopolysaccharide (LPS) will be recognized by toll-like receptor of immune cells, inducing inflammation and interfering with insulin receptor function [110]. Apart from it, Qi et al. demonstrated that bile acid metabolism was also involved in the gut microbiota alterations of PCOS individuals, as shown by an increase in *Bacteroides vulgatus* and a decrease in certain bile acids [28]. The brain-gut axis may be another potential two-way communication pathway between the gastrointestinal system and the central nervous system in PCOS that regulates appetite and energy metabolism [103, 111]. Liu et al. reported that several gut-brain peptides declined in PCOS patients when compared to healthy population, which displayed a negative correlation with clinical parameters [103]. Some researchers concurred with their conclusions while some other studies considered there were no differences in ghrelin levels between the PCOS group and the controls [112–114]. Although the current studies are still not sufficient and explicit, these gastrointestinal hormones are likely to be a link during the development of PCOS.

### IL-22 and gut mucosal immunity

Studies have elucidated that IL-22 exhibits unique properties of enhancing antimicrobial defense and tissue regeneration in different epithelial cells [41]. Deterioration of mucosal defense often occurs in obese mice, in turn, mice with mucosal immune deficiency also develop metabolic complications [115, 116]. For the lack of IL-22 induction, obese mice underperformed in response to infection and immune challenge, manifesting more severe gut barrier impairment, systemic infection and higher mortality [22]. And treatment with exogenous IL‑22 had the capacity to rescue the features. The same team also found the deficiency in IL-22 induction was due to the failure of the ILC activation mediated by IL-23 [22]. Although the specific mechanism is still unclear, it implies that IL-22 plays a key role in the preservation of intestinal epithelial barrier, thus preventing the leakage of LPS into circulation.

### IL-22 and gut microbiota

Expanding studies have supported a regulatory function for IL-22 in the gut microbiota and commensals but primarily in inflammatory and autoimmune diseases [117–119]. Wang et al. found IL-22-Fc administration in DIO mice noticeably reversed the decreased ratio of *Bacteroidetes* to *Firmicutes* that was found in obese models [22, 120]. However, the modification of bacterial compositions cannot be transferred to HFD mice in the co-housing experiment. [22] Wang et al. inferred that that IL-22 might not directly modify the microbiota in obese mice, and the positive outcomes were probably the consequence of comprehensive modulation of the systemic metabolic syndrome [22]. A new study demonstrated that IL-22 was associated with multiple gut microbiota, metabolites, and sexual hormones [99]. Moreover, they revealed that intervention with an engineered probiotic microbe could restore the decreased serum IL-22 levels in mice with PCOS, indicating that IL-22 might act as a crucial role of communication between the gut and ovary. This effect was further confirmed by administrating IL-22 inhibitor, αIL-22 [99]. αIL-22 exacerbated a number of symptoms and induced mitochondrial injury in granulosa cells of PCOS mice, which could be partially reversed by fecal transplantation of engineered probiotics [99].

### IL-22 and bile acid

Qi et al. reported that transplanting gut microbiota from PCOS patients, particularly *Bacteroides vulgatus*, could establish the PCOS-like phenotypes in mice coupled with the decrease in IL-22 whereas IL-22 administration could improve the symptoms [28]. Additionally, the bile acid metabolism pathway also regulated the IL-22 production in PCOS-like rodents. After administration of certain bile acid, the intestinal and serous IL-22 levels both elevated. Correspondingly, the benefits of bile acids diminished in mice lacking IL-22R [28]. Gao et al. also confirmed that the upregulation of bile acid profiles correlated positively with the enhance of serous IL-22 concentration in PCOS rats [29]. Another recent study reported that supplementation of a sort of yogurt could modulate IL-22 level in serum, alter microbial composition, and modify the profile of bile acid [121]. Mechanistically, Qi et al. reported that glycodeoxycholic acid (GDCA) could promote ILC development and IL-22 production through enhancing GATA3 pathway [28]. In summary, a growing corpus of research has illustrated that orienting the bile acid-IL-22 axis may represent a promising strategy for the treatment of PCOS. The work of Qi et al. has taken the initiative to shed light on the relationship between bile acid and IL-22 and left a vast space for more exploration in this field.

### IL-22 and gut-brain axis

Apart from gut mucosal protection and metabolic benefits, IL-22Fc will multiply an anorectic gut hormone called peptide YY (PYY) which could be another way to regulate body weight [22]. Additionally, IL-22 corrected an altered context of oxidative and ER stress in intestinal goblet cells and enteroendocrine cells which could secrete gut-brain peptides such as PYY [23]. However, in the pair-feeding experiment, restricted food consumption did not play a role in improving obesity, which suggested that reduced food intake might not be a primary impact on metabolism but rather an additional benefit.

## IL-22 and hepatic steatosis

NAFLD, a prevalent chronic liver disease, affects a large proportion of the global population [122, 123]. The incidence of NAFLD is 35-70% in PCOS patients, with a higher risk of more than two-fold relative to non-PCOS women [63, 124–127]. Comparatively, an elevated prevalence of PCOS has also been observed in NAFLD women of child-bearing age which implies the further interplay between the two diseases [128]. The simultaneous occurrence of the two diseases is not a mere coincidence as they share similar signs and symptoms like visceral obesity, IR, chronic low-grade inflammation, and hyperandrogenemia. The insulin-resistant adipose tissue in PCOS individuals can induce lipolysis and determine an increase of free fatty acids transportation to the liver, thus leading to hepatic fat accumulation [129]. Hyperandrogenemia not only exerts as a mediator between IR and NAFLD, but also promotes the formation of a steatogenic and proapoptotic environment which exacerbates the burden and damage of hepatocytes in PCOS [130–133]. Recently, accumulative evidence has also indicated that gut microbiome dysbiosis, which interacts closely with inflammation, may also be a link in the maintenance of PCOS and NAFLD [58, 134]. Other plausible mechanisms involve the disturbance in adipocytokine secretion, mitochondrial dysfunction, and genetic susceptibility [135–144].

### IL-22 activates STAT3 in the liver

According to the research, STAT3 signaling pathway plays an essential part in the pathophysiology of hepatic steatosis [145–147]. In the mice lack of hepatic STAT3, there was accumulation of triglyceride content and hepatic lipogenic gene expression [145]. Variants of human STAT3 genes were also relevant to NAFLD [147]. In both human hepatoma cell line HepG2 and in the mouse liver, Yang and colleagues found that the rmIL-22 could strongly activate STAT3 signaling, depending on the STAT3-binding tyrosine residues in IL-22R1 [25]. Wang et al. showed similar outcomes when administrating IL-22Fc [22].

It has also been demonstrated that the activation of the STAT3 signaling pathway inhibits lipogenesis and gluconeogenesis in the liver [145]. Studies declared that short-term rmIL-22 supplementation downregulated the gene expression of lipogenesis including critical enzymes for cholesterol and triglyceride synthesis as well as lipogenic transcription factors in both HFD rats and even normal rats [22, 25]. Though the biomarkers level in serum showed no difference, the hepatic cholesterol and triglyceride were declined [25]. Park et al. have demonstrated that IL-22 treatment dramatically suppressed gluconeogenic gene expression, which was also partially mediated by the activation of adenosine monophosphate-activated protein kinase (AMPK) [82]. Besides, long term rmIL-22 administration also suppressed TNF-α signaling pathway and the expression of genes implicated in the development of hepatic steatosis [25]. Of note, the levels of alanine transaminase (ALT) and aspartate aminotransferase (AST) were improved by IL-22 treatment as well, suggesting that IL-22 has a hepatoprotective effect [25]. However, in the mice with genetic overexpression of IL-22 (600pg/ml), Park et al. found no differences in levels of serous ALT, hepatic triglyceride (TG), and hepatic steatosis when compared to controls [82].

### IL-22 improves the oxidative stress in NAFLD

IL-22 is a potent up-regulator of antioxidant enzymes, such as metallothionein (MT)1 and MT2 [148]. In NASH mice, Hwang et al. illustrated that IL-22Fc decreased ROS levels and ROS-induced kinases phosphorylation, thus improving oxidative stress in hepatocytes [24]. Enhancing MT1 and MT2 also inhibited subsequent apoptotic signaling and the release of inflammatory extracellular vesicles (EVs) [24]. The absence of MT1 and MT2 weakened anti-inflammatory and anti-fibrotic effects of IL-22Fc in NASH mice models [24].

### The balance of IL-17 and IL-22 in the liver

A recent study shows that the infiltration of activated CD4 + cells in the liver, specifically Th17 and Th22 cells, can have a significant impact on the development of NASH in mice [149]. This study pointed that IL-17 played as a culprit in exacerbating hepatocyte lipotoxicity via the activation of the JNK pathway whereas IL-22 rescued this toxic condition through PI3K-mediated inhibition of JNK [149]. However, they also demonstrated that IL-17 nullified the protective effect of IL-22 [149]. The NASH progression was suspended in vivo by Th22 cells infiltration only in the absence of IL-17, featuring less TG content, JNK suppression and AKT activation [149]. Furthermore, IL-22 promoted the recruitment of Th17 cells in the presence of IL-17, which may exacerbate hepatic fibrosis [149, 150]. IL-22 seems to serve a dual function depending on the duration of inflammation and the intrahepatic milieu in vivo [151], however, the exogenous IL-22 as a therapeutic agent shows positive effects. It is still promising in NAFLD for its beneficial in inhibiting lipid accumulation, preserving liver function, and promoting hepatocyte proliferation and survival [25, 151, 152].

## Disscussion

Emerging advantages of IL-22 administration have been presented in different models including restoration of insulin sensitivity and glucose tolerance, resolution of inflammatory status and body weight, reconstruction of gut flora community and gut barrier, as well as reduction of hepatic fat deposition and stress injury [22, 23, 27, 28]. Accumulating knowledge of IL-22 in the attempts of treating PCOS has been gained in recent years [22, 23, 27, 28]. However, several questions are noteworthy.

First, endogenous IL-22 seems to show little effect on some diseases while it appears to have regulatory effects on PCOS. Two groups respectively demonstrated that endogenous IL-22 had no effect on the development of metabolic disorders in HFD mice [22, 82]. And IL-22-deficient mice exhibited no differences of metabolic manifestations from wild-type littermates [22]. Even in vivo, the benefits will be neutralized by other cytokines or be overwhelmed by exogenous stressors [23, 149]. The reason may be that juvenile mice without inflammatory stimulation usually holds a low level of endogenous IL-22 as Sabat concluded [98], or the efficacy of IL-22 may be restricted by the comprehensive microenvironment. However, in several studies of PCOS models, researchers have reported that various interventions can alleviate the typical symptoms by modulating the biological activity of endogenous IL-22 [28, 99, 100, 153]. Therefore, there may be disease-specificity and tissue-specificity in the role of IL-22, but the precise mechanism requires further investigation.

Second, the alteration of IL-22 levels is still confounding in rodents and humans. A majority of existing data on PCOS models and individuals indicates a consistent decline in IL-22 level, which may serve as a solid foundation for future study [27–29, 99, 121]. However, the IL-22 level varies in a number of obesity models and patients [22, 82, 95, 96]. Although illuminating explanations for different inflammatory conditions have been presented, the doubts remain. To better develop novel treatment models, we require additional clinical data and fundamental research to comprehend the alterations and mechanisms of IL-22 in PCOS and other metabolic diseases.

Third, the precise concentration of IL-22 in sure of safety and effectiveness remains to be confirmed. Researchers have indicated that appropriate exogenous IL-22 concentrations could provide substantial protection. Whereas considerable high concentration of IL-22 probably leads to cachexia and certain relatively high concentration appears to play no role in obese mice [82]. Besides, high local levels of IL-22 show pathological effects in some other diseases. It can promote the psoriasis pathological progression, intestinal proliferation and even neoplasia [154, 155]. Despite not being the onset of liver cancer, IL-22 may stimulate the growth of existing hepatic tumors via STAT3 activation [148, 156, 157]. Also, IL-22 indirectly promotes the progression of chronic viral hepatitis [150]. However, Hasnain et al. observed no changes in the histology or morphology of skin or gut with short-term and sporadic administration [23]. To assure the safety and efficacy of IL-22, it is still necessary to determine an optimal administration protocol. Before it can be implemented in clinical practice as an alternative approach for PCOS, further explorations in the field of regulating metabolism and immunity by IL-22 remain to be consolidated.

## Conclusion

In conclusion, IL-22 tends to be a potential therapy method for PCOS patients in the future. However, we must take into account that the function of IL-22 varies depending on the administration dosage and the specific tissue. In order to mitigate the adverse effects, a suitable and targeted mode of delivery and dosage should be considered when developing novel medications for PCOS. Although the modulation of systemic metabolic disorders and inflammation is crucial for PCOS patients, we should also investigate deeper into how IL-22 will regulate other typical phenotypes of PCOS.

## Data Availability

Not applicable.

## Abbreviations

PCOS: polycystic ovary syndrome
IL-22: interleukin-22
IR: insulin resistance
MS: metabolic syndrome
T2DM: type 2 diabetes mellitus
COCP: combined oral contraceptive pill
IFN: interferon
NKT: natural killer T
ILCs: innate lymphocyte cells
Th17: T-helper 17
Th22: T-helper 22
IL-22R: interleukin-22 receptor
IL-22BP: interleukin-22-binding protein
NAFLD: nonalcoholic fatty liver disease
NASH: nonalcoholic steatohepatitis
GLUT-4: glucose transporter type 4
SHBG: sex hormone binding globulin
T: testosterone
HFD: high fat diet
ER: endoplasmic reticulum
STAT1: signal transducer and activator of transcription 1
JAK1: Janus kinase 1
BAT: brown adipose tissue
IL-22Fc: IL-22 fragment crystallizable
AKT: protein kinase B
DIO: diet-induced obese
JNK: c-Jun kinase
TNF-α: tumor necrosis factor-α
CRP: C-reactive protein
SCFAs: short-chain free fatty acids
LPS: lipopolysaccharide
GDCA: glycodeoxycholic acid
TUDCA: tauroursodeoxycholic acid
PYY: peptide YY
AMPK: adenosine monophosphate-activated protein kinase
ALT: alanine transaminase
AST: aspartate aminotransferase
MT: metallothionein
EVs: extracellular vesicles
OC: oral contraceptive
rm IL-22: recombinant mouse IL-22
TG: triglyceride
